# Epidemiological Characteristics of 101,471 Patients Hospitalized with Chronic Obstructive Pulmonary Disease (COPD) in Poland in 2019: Multimorbidity, Duration of Hospitalization, In-Hospital Mortality

**DOI:** 10.3390/arm91050029

**Published:** 2023-09-20

**Authors:** Mateusz Jankowski, Bogdan Bochenek, Joanna Wieczorek, Mariusz Figurski, Marta Gruszczyńska, Paweł Goryński, Jarosław Pinkas

**Affiliations:** 1 School of Public Health, Centre of Postgraduate Medical Education, 01-826 Warsaw, Poland; 2 Institute of Meteorology and Water Management-National Research Institute, 01-673 Warsaw, Poland; 3 Department of Population Health Monitoring and Analysis, National Institute of Public Health NIH—National Research Institute, 00-791 Warsaw, Poland

**Keywords:** chronic obstructive pulmonary disease, COPD, national registry, epidemiology, hospitalization, clinical characteristics, multimorbidity, in-hospital mortality, Poland

## Abstract

**Highlights:**

**What are the main findings?**

- The presence of comorbidities was reported in 89.3% of patients hospitalized with COPD, the average duration of hospitalization was 9.4 days (54.5% of patients hospitalized with COPD were hospitalized for up to 7 days), and the in-hospital mortality rate was 6.8%.
- Older age, presence of cardiovascular diseases, genitourinary disorders, or digestive disorders were significantly associated with a higher risk of in-hospital death.

**What is the implication of the main finding?**

- Findings from this nationwide registry-based survey may be used by clinicians during the development of guidelines on COPD management.
- Efforts should be made to develop models for assessing and predicting the impact of air pollution and meteorological factors on hospital admissions of patients diagnosed with COPD.

**Abstract:**

Chronic obstructive pulmonary disease (COPD) is a common lung disease. There is a limited amount of nationwide data on COPD patients in Poland. This study aimed to characterize patients hospitalized with COPD in Poland in 2019 as well as to identify factors associated with the risk of in-hospital death and prolonged hospitalization among patients with COPD. This study is a retrospective database analysis. Data on patients hospitalized with COPD in Poland were obtained from the Nationwide General Hospital Morbidity Dataset. Data on all adults aged ≥40 years with a diagnosis of COPD from a physician (J44 code) were included in the analysis. Data were analyzed separately for patients hospitalized due to COPD (primary diagnosis) and patients with COPD as a comorbidity (secondary diagnosis). Completed medical records were available for 101,471 patients hospitalized with COPD (36.9% were females). Of those, 32% were hospitalized due to COPD. The mean age was 71.4 ± 9.7 years. The mean duration of hospitalization was 9.4 ± 11.4 days (median 7 days). Most of the COPD patients (89.3%) had at least one comorbidity. The in-hospital mortality rate was 6.8%. Older age, presence of cardiovascular diseases, and diseases of the genitourinary system (*p* < 0.05) were the most important factors associated with the risk of in-hospital death among patients hospitalized due to COPD.

## 1. Introduction

Chronic obstructive pulmonary disease (COPD) is a common lung disease characterized by persistent respiratory symptoms and airflow limitation [1,2,3]. COPD often presents with breathing difficulty, cough, mucus/sputum production, and wheezing [3,4]. COPD diagnosis is based on respiratory function tests, mostly spirometry [1,2]. Long-term exposure to irritating gases or particulate matter (especially tobacco smoke) is the most common risk factor for COPD [5,6,7]. Most COPD cases can be prevented by reducing the prevalence of tobacco use, smoke-free policy implementation, and limiting exposure to indoor and outdoor pollutants [2]. COPD patients are at higher risk for cardiovascular disease, lung cancer, skeletal muscle dysfunction, metabolic syndrome, osteoporosis, and depression [2,8].

COPD is a progressive condition that becomes more severe with time [9]. The natural course of COPD is aggravated by episodes of exacerbations defined as an acute worsening of respiratory symptoms requiring a change in treatment [10]. COPD treatment aims to reduce symptoms, frequency, and severity of exacerbations as well as improve health status and quality of life [1,2,10]. Management of COPD includes both pharmacological and non-pharmacological interventions [1,2,11]. Inhaled bronchodilators and corticosteroids are the most common drugs for the management of stable COPD [2,11]. Patients with COPD exacerbation may require additional treatment, including antibiotics and adjunct therapies [2]. Non-pharmacological treatment of COPD includes, e.g., smoking cessation, vaccinations, nutritional support, pulmonary rehabilitation programs, oxygen therapy, and ventilatory support [2]. 

COPD can be effectively managed in outpatient settings, especially in primary care [2,12]. However, patients with COPD exacerbation may require hospitalization [10,11,12]. Hospitalizations related to COPD exacerbations and complications generate substantial socioeconomic costs [13,14]. In addition, some patients with COPD may be admitted to the hospital, e.g., for the diagnosis of comorbidities. It is estimated that between 3% and 20% of patients with COPD require at least one hospital admission per year [15]. COPD hospitalizations account for 33–57% of the total costs of COPD management [15]. 

COPD is the third leading cause of death worldwide [2]. The global prevalence of COPD is estimated at 10.3%, translating to 392 million people worldwide [16]. Epidemiological estimates indicate that around two million people suffer from COPD in Poland [17]. However, due to low public awareness of COPD in Poland, a significant proportion of patients may be not aware of the disease [18]. In Poland, most COPD cases are managed in outpatient settings (mostly in primary care). Most of the data on COPD patients in Poland are limited to local studies or some regional data [19,20,21]. There is a lack of nationwide data on COPD patients in Poland. Epidemiological data on patients hospitalized with COPD (especially multimorbidity and duration of hospitalization and its outcome) can provide clinicians with information useful in developing national guidelines and recommendations for the treatment of COPD and its exacerbations. Data on the duration of hospitalization, admission wards, and in-hospital mortality may provide information on pulmonary care in Poland and the need for organizational changes to better adapt the health care system to the needs of patients with respiratory diseases. Nationwide data on COPD patients may contribute to health policy planning and support the development of clinical guidelines [22]. 

Therefore, this study aimed to characterize patients hospitalized with COPD in Poland in 2019 as well as to identify factors associated with the risk of in-hospital death and prolonged hospitalization among patients with COPD.

## 2. Materials and Methods

### 2.1. Study Design and Data Source

This study is a retrospective database analysis. Data on patients hospitalized with COPD in Poland were obtained from the Nationwide General Hospital Morbidity Dataset [23], managed by the National Institute of Public Health National Institute of Hygiene—National Research Institute (NIZP PZH-PIB) [23,24]. All hospitals in Poland (except the psychiatric hospitals) are obligated to report all hospitalizations (using a dedicated form) to the NIZP PZH-PIB as a part of public statistics [23]. Reporting forms contains anonymized information about the patient’s hospitalization (similar to a hospital discharge report), including gender, age, characteristics of the hospital, place of residence, admission mode (emergency/scheduled), duration of hospitalization, primary diagnosis (cause of admission), secondary diagnosis (comorbidities), and outcome of hospitalization (in-hospital death or discharge) [23]. Data on patients’ medical conditions are based on a medical diagnosis and coded by physicians using the 10th revision of the International Statistical Classification of Diseases (ICD-10) [25]. A detailed description of the data collection process has been described in previous papers [24,26]. 

Data on all patients hospitalized with COPD in 2019 for whom complete medical information was available were included in the analysis. The data set used in the study is anonymous and it was not possible to identify multiple hospitalizations of the same patient. Each admission of a patient with COPD to the hospital was considered a separate hospitalization. Hospitalization lasts from admission to discharge (or in-hospital death). Clinical guidelines indicate the need to limit rehospitalization of COPD patients and develop ambulatory care for COPD patients [2]. 

### 2.2. Measures

#### 2.2.1. COPD Diagnosis

Patients diagnosed with COPD were identified using ICD-10 code J44 (including J44.0–J44.9) [25]. Based on the usual onset of COPD (40 years and over), only data on patients aged 40 and over were included in the analysis. Patients diagnosed with J44 before the age of 40 (662 records in the primary dataset) were not included in this analysis. Acute COPD exacerbation was based on the ICD-19 code J44.1 [25]. COPD with acute lower respiratory infection was based on the ICD-19 code J44.0 [25]. ICD coding is the most common methodological approach for the identification of COPD patients through national registries and datasets [27].

#### 2.2.2. Multimorbidity

Comorbidities were identified using ICD-10 codes [25]. Diagnosis of at least one disease (ICD-10 code) from the following groups was considered as the presence of comorbidities (at least one disease) from a given group: cardiovascular diseases (I00–99); endocrine, nutritional, metabolic diseases (E00–90); diseases of the genitourinary system (N00–99); diseases of the digestive system (K00–93); diseases of the skin and subcutaneous tissue (L00–99); diseases of the musculoskeletal system and connective tissue (M00–99); and diseases of the nervous system (G00–99) [25]. Hypertension was identified using ICD-10 codes I10–15, heart failure was identified using ICD-10 code I50, diabetes (regardless of the type) was identified using ICD-10 codes E10–14, and chronic kidney failure was identified using ICD-10 code N18 [25].

#### 2.2.3. Cause of Admission

Based on the primary diagnosis (cause of admission listed in reporting form), patients were divided into two groups: those hospitalized due to COPD (COPD as a primary diagnosis), and patients hospitalized due to other medical conditions, but also diagnosed with COPD as a comorbidity.

#### 2.2.4. Type of Admission

Based on the mode of admission to the hospital specified in the reporting form, hospitalizations were divided into emergency admissions (urgent health needs) and scheduled admission (with referral). First-admission ward was identified using a code defined by the National Health Fund (public payer in Poland) [28].

#### 2.2.5. Duration of Hospitalization

The duration of hospitalization was calculated based on admission and discharge dates. Two scenarios of prolonged hospitalization were analyzed. In the first scenario, hospitalizations longer than the median duration of hospitalization were classified as prolonged (>7 days). In the second scenario, hospitalizations longer than the average duration of hospitalization were classified as prolonged (>10 days).

#### 2.2.6. In-Hospital Mortality

In-hospital mortality was defined as the percentage of fatal cases out of all hospitalizations. In-hospital mortality rates were calculated separately for patients hospitalized due to COPD and patients with COPD as a comorbidity. 

### 2.3. Data Analysis

The data were analyzed with SPSS v. 28 (IBM, Armonk, NY, USA). The distribution of the categorical variables was shown by frequencies and proportions. Statistical testing to compare categorical variables was completed using the independent samples chi-square test. Data were presented separately for hospitalizations due to COPD and hospitalizations due to other conditions but with COPD as a comorbidity. In this study, each hospital admission was considered as an independent observation.

Multivariable logistic regression analyses were carried out to identify factors associated with the risk of in-hospital death. Age, gender, and the presence of the 4 most common groups of comorbidities (cardiovascular diseases; endocrine, nutritional, and metabolic diseases; disease of the genitourinary system; and disease of the digestive system) were considered as independent variables. Moreover, multivariable logistic regression analyses were carried out to identify factors associated with the risk of prolonged hospitalization (two scenarios: (1) >7 days; (2) >10 days). Age, gender, presence of at least one comorbidity, and type of admission were considered independent variables. The strength of the association was measured by the odds ratio (OR) and 95% confidence intervals (CI). The statistical significance level was set at *p* < 0.05.

## 3. Results

### 3.1. Clinical Characteristics of Patients Hospitalized with COPD

Completed medical records were available for 101,471 patients hospitalized with COPD (Table 1). Of those, 32% were hospitalized due to COPD, and 68% were hospitalized due to other medical conditions but were also diagnosed with COPD as a comorbidity (Table 1). The mean age was 71.4 ± 9.7 years (median 71 years; range 40–105 years). Most of the patients hospitalized with COPD were males (63.1%). Over two-thirds of hospitalizations were emergency admissions (67.1%). During hospitalization, 81.1% of patients with COPD stayed in only one ward, and the most common first-admission ward was the internal medicine department (30.7%). Among the patients, 29.4% stayed in the tuberculosis and/or lung diseases department, and 15% were admitted to the emergency department (Table 1). The mean duration of hospitalization was 9.4 ± 11.4 days (median 7 days; range 0–564 days). Among the patients, 5.2% were hospitalized for less than 24 h, and 54.5% of patients hospitalized with COPD were hospitalized for up to 7 days. Out of all patients hospitalized with COPD, the in-hospital mortality rate was 6.8% (Table 1). On admission, 21% of patients were diagnosed with acute COPD exacerbation (J44.1), and 12.2% had COPD with acute lower respiratory infection (J44.0). The remaining patients presented with other or unspecified COPD on admission. There were statistically significant differences (*p* < 0.001) between patients hospitalized due to COPD and patients hospitalized due to other medical conditions but with COPD as a comorbidity (Table 1).

### 3.2. Seasonality of Hospital Admissions of Patients Diagnosed with COPD

**Figure 1:**
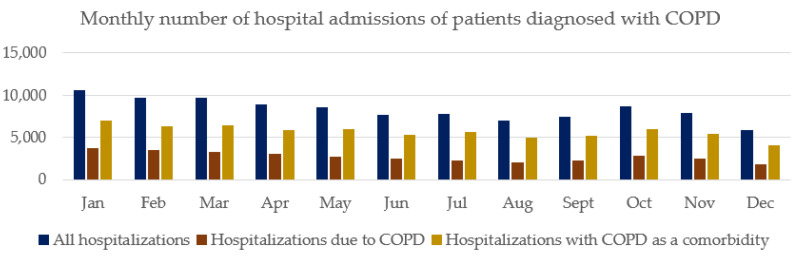
Seasonality of hospital admissions of patients diagnosed with COPD.

There were differences in the monthly number of hospital admissions of patients diagnosed with COPD (Figure 1). Among all patients hospitalized with COPD, the highest number of hospital admissions was observed in January and October. Among patients hospitalized due to COPD, there were two peaks of hospital admissions: the first observed between January and February and the second between October and November. The lowest number of hospital admissions was observed in December (Figure 1).

### 3.3. Multimorbidity in Patients Hospitalized with COPD

Out of 101,471 patients hospitalized with COPD, 89.3% had at least one comorbidity. Among patients hospitalized due to COPD, the presence of comorbidities was reported in 66.5% of patients. Most of the patients with COPD (60.5%) had also at least one cardiovascular disease (Table 1). Over one-quarter of patients had hypertension (28.6%) or heart failure (27.0%). Endocrine, nutritional, and metabolic diseases were the second most common (18%) group of diseases diagnosed in patients with COPD. Diabetes was diagnosed in 10.9% of patients diagnosed with COPD. Almost one-tenth of patients with COPD were also diagnosed with diseases of the genitourinary system (9.0%), 3.4% had chronic kidney failure, 8.8% had lung cancer, 6.1% of patients had diseases of the digestive system (Table 1). Other groups of diseases were reported in less than 5% of patients. 

### 3.4. Clinical Characteristics of Patients Hospitalized with COPD by Type of Admission

There were significant differences in the characteristics of the patients hospitalized with COPD by type of admission (Table 2). Among patients hospitalized due to COPD admitted in emergency mode, 58.6% had acute COPD exacerbation (J44.1) or COPD with an acute lower respiratory infection (J44.0) (Table 2). Over 39% of patients admitted urgently to the hospital stayed in the internal medicine department. Most of the scheduled admissions (>95%) were limited to one hospital ward (Table 2). Among patients hospitalized due to COPD, in-hospital mortality rate was 5.5% for emergency admissions and 1.4% for scheduled admissions (*p* < 0.001). Over half of the patients with COPD admitted urgently were hospitalized for up to 7 days. Of patients hospitalized with COPD on schedule mode, 57% were hospitalized for more than 7 days. Among patients hospitalized due to other medical conditions, but also diagnosed with COPD as a comorbidity, the in-hospital mortality rate was 10.9% for emergency admissions and 2.7% for scheduled admissions (*p* < 0.001). Details are presented in Table 2.

### 3.5. Gender Differences in Characteristics of Patients Hospitalized with COPD

Both among patients hospitalized due to COPD and patients hospitalized due to other diseases but with COPD as comorbidity, gender differences in age, number of hospital wards, first-admission hospital ward, duration of hospitalization, and prevalence of selected comorbidities were observed (Table 3). There were no differences in COPD diagnosis during the admission and in-hospital mortality by gender (*p* > 0.05). Details are presented in Table 3.

### 3.6. Factors Associated with the Risk of In-Hospital Death and Prolonged Hospitalization

In multivariable logistic regression models, among all patients hospitalized with COPD in Poland, older age (*p* < 0.05), the presence of cardiovascular disease (OR: 1.87; 95%CI: 1.77–1.98; *p* < 0.001), the presence of disease of the genitourinary system (OR: 1.55; 95%CI: 1.44–1.67; *p* < 0.001), and the presence of disease of the digestive system (OR: 1.17; 95%CI: 1.44–1.67; *p* < 0.001) were associated with higher risk of in-hospital death (Table 4). In patients hospitalized due to COPD only age 60 and over (*p* < 0.05), the presence of cardiovascular disease (OR: 1.94; 95%CI: 1.73–2.18; *p* < 0.001) and the presence of disease of the genitourinary system (OR: 1.30; 95%CI: 1.08–1.57; *p* = 0.01) were associated with a higher risk of in-hospital death (Table 4).

Age 60 years and over, female gender, presence of at least one comorbidity, and emergency admission mode were significantly associated (*p* < 0.05) with a higher risk of hospitalization for over 7 days, both among all patients hospitalized with COPD as well as those admitted to the hospital due to COPD (Table 5). Age 60 years and over, female gender, presence of at least one comorbidity, and scheduled admission mode were significantly (*p* < 0.05) associated with a higher risk of hospitalization for over 10 days (Table 5), both among all patients hospitalized with COPD as well as those admitted to the hospital due to COPD.

## 4. Discussion

This national-registry-based study provided data on the clinical characteristics of over 100 thousand patients hospitalized with COPD in Poland in 2019. COPD was the primary diagnosis (cause of hospitalization) for almost one-third of patients (32%), and over two-thirds of hospital admissions (67.1%) were emergency admissions. Of all patients hospitalized with COPD, 81.1% stayed only in one hospital ward. This study also presented data on multimorbidity among patients with COPD, and the presence of comorbidities was reported in 89.3% of patients hospitalized with COPD. The average duration of hospitalization was 9.4 days, and 54.5% of patients hospitalized with COPD were hospitalized for up to 7 days. Age, gender, presence of comorbidities, and type of admission were significantly associated with the risk of prolonged hospitalization. The in-hospital mortality rate was 6.8%, wherein a lower rate was observed among patients admitted to the hospital due to COPD (4.4%) rather than patients hospitalized due to other medical conditions but with COPD as a comorbidity (7.9%). Older age, presence of cardiovascular diseases, genitourinary disorders, or digestive disorders were significantly associated with a higher risk of in-hospital death.

The global burden of COPD is increasing [2,16]. COPD is the third leading cause of death worldwide [2]. In highly developed countries like Poland, most COPD cases are the result of tobacco use [6,7]. In highly developed countries like Poland, most COPD cases are the result of tobacco use [6,7]. It is estimated that over one-fourth of adults in Poland smoke regularly [29]. Due to the high prevalence of tobacco use in Poland [29], the COPD burden in Poland is expected to increase in the next decades. Despite the high health, social, and economic burden of COPD in Poland [30,31], there is a limited amount of epidemiological data on COPD in Poland [7,10,32,33]. Moreover, numerous studies reported that COPD is remarkably underdiagnosed in Poland [32,33,34,35]. Data on multimorbidity in COPD patients may be used to inform physicians about the most common comorbidities that coexist with COPD in Poland and indicate priority populations for COPD screening.

In this study, data on patients hospitalized with COPD were presented separately for those hospitalized due to COPD (primary diagnosis) and those hospitalized due to other diseases but with diagnosis of COPD as a comorbidity. Data were presented separately to provide more precise data on patients hospitalized with COPD in Poland. COPD patients are at higher risk of other comorbidities like cardiovascular diseases, metabolic syndrome, and osteoporosis [2,8], so multimorbidity (especially the coexistence of respiratory diseases and cardiovascular diseases) may lead to the fact that COPD was classified as a secondary diagnosis in 68% of patients.

Most of the patients hospitalized with COPD were admitted urgently. In patients hospitalized due to COPD, 72.9% of hospitalizations were emergency admissions. COPD exacerbation is the most common cause of hospitalization due to COPD [10,11,12]. Findings from this study suggest that physicians may lack knowledge in the diagnosis of COPD exacerbations. Based on the ICD-10 codes used for the COPD diagnosis during admission, only 50.5% of patients hospitalized due to COPD in Poland were diagnosed with acute COPD exacerbation (J44.1; 32.8%) or COPD with an acute lower respiratory infection (J44.0; 17.7%). Based on the clinical course of COPD [2], we can hypothesize that most of the hospitalizations due to COPD were related to COPD exacerbation. As this study is based on the most important public registry on hospitalizations in Poland (Nationwide General Hospital Morbidity Dataset), findings from this study underline the need to educate physicians on COPD exacerbation diagnosis as well as the data reporting for public statistics and epidemiological reports. Moreover, there is a need for further research to determine the impact of environmental factors on the periodic and spatial variability of COPD exacerbation in the Polish population [36].

Most of the patients hospitalized with COPD were first admitted to the internal medicine department (30.7%) and 29.4% were admitted to the tuberculosis and/or lung diseases department. The percentage of patients first admitted to the emergency department was two times higher among those hospitalized due to COPD compared to those hospitalized due to other conditions but with COPD as a comorbidity (22.9% vs. 11.3%). We can hypothesize that emergency department admissions were mostly related to acute COPD exacerbations [10]. This study also suggests that there is a shortage of tuberculosis and/or lung disease departments in Poland. Due to the growing burden of COPD in Poland, there is a need to develop regional respiratory disease centers and encourage young doctors to train in the field of lung diseases. Moreover, postgraduate training on COPD management targeted to general practitioners and internal medicine specialists should be developed to improve the effectiveness of COPD management in ambulatory settings. 

Hospitalizations of patients with COPD pose a significant economic burden for national health systems [13,14]. The duration of hospitalization influences the economic costs as well as the healthcare resource utilization. In this study, the mean duration of hospitalization was 9.4 days, wherein most of the patients (54.5%) were hospitalized for up to 7 days. This observation is comparable to findings from the population-based studies in Italy and Catalunya, Spain, where the mean length of hospitalization due to COPD exacerbation was 9.95 ± 8.69 days [37] and 7.05 ± 5.79 days respectively [38]. However, it should be noted that, in this study, all hospitalizations with COPD were analyzed, compared to only COPD exacerbations in the studies from Italy and Catalunya, Spain [37,38]. In this study, age 60 years and over, female gender, presence of at least one comorbidity, and emergency admission mode were associated with the risk of hospitalization for more than 7 days. Moreover, age 60 years and over, female gender, presence of at least one comorbidity, and scheduled admission were associated with the risk of hospitalization for more than 10 days. This observation may have a practical implication for clinicians to predict the duration of hospitalization among patients with COPD admitted to the hospital. Most of the patients hospitalized with COPD were males, but females were at higher risk of prolonged hospitalization. 

Multimorbidity is a significant clinical issue, associated with elevated risk of death, disability, and poor quality of life, as well as adverse drug events [39]. This registry-based study showed that 89.3% of patients hospitalized with COPD had comorbidities, wherein cardiovascular disease where the most common (60.5% of patients). This observation is in line with the previously published data. Cardiovascular diseases are perceived as the most important comorbidities in COPD that influence the prognosis of patients [40,41]. Almost one-fourth of COPD patients were also diagnosed with metabolic diseases (mostly diabetes). Type 2 diabetes is a risk factor for severe COPD [42]. Moreover, there is a scientific debate on the potential link between COPD and type 2 diabetes [43]. COPD was found to be associated with chronic kidney disease in numerous studies [44,45]. In this study, diseases of the genitourinary system were the third most common group of comorbidities among patients with COPD. This observation is in line with previous epidemiological studies on COPD and the risk of developing kidney diseases [44,45]. Data on multimorbidity among patients hospitalized with COPD may be used by clinicians and public health specialists to develop clinical guidelines on COPD management in Poland and models of managed care in COPD.

In this study, in-hospital mortality was also assessed. Among all patients hospitalized with COPD, the in-hospital mortality rate was 6.8%, compared to 4.4% among patients admitted to the hospital due to COPD and 7.8% among patients with COPD but admitted to the hospital due to other medical conditions. Among patients hospitalized with COPD, in-hospital mortality rates reported in the literature vary from 2.5% to 30%, depending on the study population and settings [46]. In the Italian population-based study, the mean in-hospital mortality was 5.3% [37], which is comparable to those observed in this registry-based study. In this study, older age, the presence of cardiovascular diseases, and diseases of the genitourinary system or digestive system were significantly associated with increased risk of in-hospital mortality among patients hospitalized with COPD. This finding indicates on groups of patients with comorbidities increase the risk of in-hospital death among COPD patients.

### 4.1. Practical Implications

Findings from this nationwide registry-based survey may be used by clinicians during the development of guidelines on COPD management. Data on patients hospitalized with COPD may indicate populations that are at higher risk of COPD exacerbation. Data on medical coding and ICD-10 codes used by physicians indicate a need to improve the quality of data reporting. This may partially result from the limited public awareness of COPD in Poland [18]. Moreover, this study points to the need to develop healthcare resources related to respiratory medicine in Poland, including infrastructure (lung diseases departments) and human resources (healthcare workers trained in respiratory medicine). The obtained results showed that COPD does not affect only the oldest population. The share of patients hospitalized due to or coexisting with COPD aged <70 years, as well as hospital deaths in younger age groups, may increase in the coming years, especially among women, which is also indicated by earlier studies [33]. This study also points to the need for reconsideration of guidelines and protocols on COPD exacerbation prevention and management. From the perspective of healthcare organization and health resource utilization, even multiple hospitalizations of the same patients pose a significant burden, so data presented in this study are targeted both to clinicians as well as public health specialists and policymakers. Early diagnosis and education about COPD should also be addressed to people of working age. Findings from this study may be used by policymakers to develop health policies related to non-communicable diseases, especially COPD. Efforts should be made to develop models for assessing and predicting the demand for medical services to optimize the operation of the existing facilities. Environmental factors may affect the seasonality of exacerbations of respiratory diseases at the national and regional levels [47]. Further studies should analyze the impact of air pollution and meteorological factors [47,48,49] on hospital admissions of patients diagnosed with COPD. 

### 4.2. Limitations

This study has limitations typical for retrospective national database analysis. Data were obtained from the public registry managed by the NIZP PZH-PIB, and the scope of analysis is limited to data available in the database. Data on the COPD severity and COPD stages (e.g., based on the Global Initiative for Chronic Obstructive Lung Disease (GOLD) or COPD assessment test (CAT)) were not available. Moreover, data on pharmacotherapy were not available. Also, data on lifestyle-related habits, including smoking history, were not recorded in the reporting form. Data on clinical conditions and diagnosis were based on ICD-10 coding. Due to the quality of data reported by physicians in Poland, the prevalence of COPD exacerbations (ICD-10 codes J44.1 and J44.0) and comorbidities may be underreported. Nevertheless, this is the first comprehensive characteristic of patients hospitalized with COPD in Poland which is based on data from the nationwide public registry.

## 5. Conclusions

In this national-registry-based study, detailed epidemiological characteristics of patients hospitalized with COPD in Poland were presented. Multimorbidity is a significant clinical issue among patients hospitalized with COPD. Most COPD patients are admitted in emergency mode and stay in only one ward during hospitalization. Age 60 years and over, the presence of cardiovascular diseases, and diseases of the genitourinary system are the most important factors associated with the risk of in-hospital death among patients admitted due to COPD. The data presented in this study may contribute to health policy planning and clinical guidelines development. 

## Figures and Tables

**Table 1:** Characteristics of the patients hospitalized with COPD in Poland in 2019.

Variable	Total Population (n = 101,471)		Patients Hospitalized due to COPD (n = 32,425)		Patients with COPD as a Comorbidity (n = 69,046)		*p*
	n	%	n	%	n	%	
**Gender**							
male	64,055	63.1	20,198	62.3	43,857	63.5	**<0.001**
female	37,416	36.9	12,227	37.7	25,189	36.5	
**Age group (years)**							
40–49	1484	1.5	471	1.5	1013	1.5	**0.003**
50–59	8468	8.3	2625	8.1	5843	8.5	
60–69	34,835	34.3	10,929	33.7	23,906	34.6	
70–79	34,545	34.0	11,252	34.7	23,293	33.7	
80+	22,139	21.8	7148	22.0	14,991	21.7	
**COPD diagnosis during the admission**							
acute COPD exacerbation (J44.1)	21,280	21.0	10,632	32.8	10,648	15.4	<0.001
COPD with acute lower respiratory infection (J44.0)	12,423	12.2	5747	17.7	6676	9.7	<0.001
**Type of hospital admission**							
emergency admission	68,103	67.1	23,654	72.9	44,449	64.4	<0.001
scheduled admission	33,368	32.9	8771	27.1	24,597	35.6	
**Number of hospital wards**							
1	82,272	81.1	25,024	77.2	57,248	82.9	<0.001
2	16,237	16.0	6823	21.0	9414	13.6	
3–5	2962	2.9	413	1.3	1766	2.6	
**First-admission hospital ward**							
internal medicine department	31,111	30.7	10,521	32.4	20,590	29.8	0.001
tuberculosis and lung diseases department	12,433	12.3	3586	11.1	8847	12.8	
lung disease department	17,336	17.1	5011	15.5	12,325	17.9	
emergency department	15,240	15.0	7419	22.9	7821	11.3	
cardiology department	3858	3.8	145	0.4	3713	5.4	
oncology department	2773	2.7	21	0.1	2752	4.0	
other type of hospital ward	18,720	18.4	5722	17.6	12,998	18.8	
**Duration of hospitalization (days)**							
0 (<24 h)	5289	5.2	1614	5.0	3675	5.3	<0.001
1–3	16,931	16.7	3944	12.2	12,987	18.8	
4–7	34,048	33.6	11,764	36.3	22,284	32.3	
8–14	28,433	28.0	9276	28.6	19,157	27.7	
15–21	10,818	10.7	4703	14.5	6115	8.9	
22–31	3249	3.2	691	2.1	2558	3.7	
32 and more	2703	2.7	433	1.3	2270	3.3	
**Comorbidities**							
cardiovascular diseases (I00–99)	61,387	60.5	15,458	47.7	45,939	66.5	<0.001
hypertension (I10–15)	28,994	28.6	8772	27.1	20,222	29.3	<0.001
heart failure (I50)	27,350	27.0	5744	17.7	21,606	31.3	<0.001
endocrine, nutritional, and metabolic diseases (E00–90)	18,305	18.0	4686	14.5	13,619	19.7	<0.001
diabetes (E10–E14)	11,051	10.9	2848	8.8	8203	11.9	<0.001
diseases of the genitourinary system (N00–99)	9105	9.0	1981	6.1	7124	10.3	<0.001
chronic kidney failure (N18)	3400	3.4	667	2.1	2773	4.0	<0.001
diseases of the digestive system (K00–93)	6229	6.1	1129	3.5	5100	7.4	<0.001
diseases of the skin and subcutaneous tissue (L00–99)	769	0.8	80	0.2	689	1.0	<0.001
diseases of the musculoskeletal system and connective tissue (M00–99)	4707	4.6	1338	4.1	3369	4.9	<0.001
diseases of the nervous system (G00–99)	2972	2.9	661	2.0	2311	3.3	<0.001
lung cancer	8965	8.8	384	1.2	8581	12.4	0.001
**In-hospital death**							
yes	6898	6.8	1411	4.4	5487	7.9	<0.001
no	94,573	93.2	31,014	95.6	63,559	92.1	

**Table 2:** Characteristics of the patients hospitalized with COPD by type of admission.

Variable	Patients Hospitalized due to COPD (n = 32,425)			Patients with COPD as a Comorbidity (n = 69,046)		
	Emergency Admission n = 23,654	Scheduled Admission n = 8771	*p*	Emergency Admission n = 44,449	Scheduled Admission n = 24,597	*p*
**Gender**	**n (%)**	**n (%)**		**n (%)**	**n (%)**	
male	14,559 (61.5)	5639 (64.3)	<0.001	28,080 (63.2)	15,777 (64.1)	0.01
female	9095 (38.5)	3132 (35.7)		16,369 (36.8)	8820 (35.9)	
**Age group [years]**						
40–49	308 (1.3)	163 (1.9)	<0.001	504 (1.1)	509 (2.1)	<0.001
50–59	1713 (7.2)	913 (10.4)		3076 (6.9)	2767 (11.2)	
60–69	7457 (31.5)	3472 (39.6)		13,879 (31.2)	10,027 (40.8)	
70–79	8259 (34.9)	2993 (34.1)		15,045 (33.8)	8248 (33.5)	
80+	5917 (25.0)	1231 (14.0)		11,945 (26.9)	3046 (12.4)	
**COPD diagnosis during the admission**						
acute COPD exacerbation (J44.1)	9122 (38.6)	1510 (17.2)	<0.001	8643 (19.4)	2005 (8.2)	<0.001
COPD with acute lower respiratory infection (J44.0)	4724 (20.0)	1023 (11.7)	<0.001	5468 (12.3)	1208 (4.9)	<0.001
**Number of hospital wards**						
1	16,543 (69.9)	8481 (96.7)	<0.001	33,716 (75.9)	23,532 (95.7)	<0.001
2	6580 (27.8)	243 (2.8)		8920 (20.1)	494 (2.0)	
3–5	531 (2.2)	47 (0.5)		1813 (4.0)	571 (2.3)	
**First-admission hospital ward**						
internal medicine department	9254 (39.1)	1267 (14.4)	<0.001	17,341 (39.0)	3249 (13.2)	<0.001
tuberculosis and lung diseases department	1867 (7.9)	1719 (19.6)		4088 (9.2)	4759 (19.3)	
lung disease department	2715 (11.5)	2296 (26.2)		5718 (12.9)	6607 (26.9)	
emergency department	7247 (30.6)	-		7689 (17.3)	-	
cardiology department	132 (0.6)	13 (0.1)		2062 (4.6)	1651 (6.7)	
oncology department	12 (0.1)	9 (0.1)		953 (2.1)	1799 (7.3)	
other type of hospital ward	2427 (10.3)	3467 (39.6)		6598 (14.8)	6532 (26.5)	
**Duration of hospitalization (days)**						
0 (<24 h)	1397 (5.9)	217 (2.5)	<0.001	1305 (2.9)	2370 (9.6)	<0.001
1–3	2862 (12.1)	1082 (12.3)		6636 (14.9)	6351 (25.8)	
4–7	9291 (39.3)	2473 (28.2)		14,807 (33.3)	7477 (30.4)	
8–14	7546 (31.9)	1730 (19.7)		14,386 (32.4)	4771 (19.4)	
15–21	1642 (6.9)	3061 (34.9)		4068 (9.2)	2047 (8.3)	
22–31	572 (2.4)	119 (1.4)		1777 (4.0)	781 (3.2)	
32 and more	344 (1.5)	89 (1.0)		1470 (3.3)	800 (3.3)	
**Comorbidities**						
cardiovascular diseases (I00–99)	11,649 (49.2)	3809 (43.4)	<0.001	31,983 (72.0)	13,956 (56.7)	<0.001
hypertension (I10–15)	6118 (25.9)	2654 (30.3)	<0.001	12,445 (28.0)	7777 (31.6)	<0.001
heart failure (I50)	4942 (20.9)	802 (9.1)	<0.001	17,816 (40.1)	3790 (15.4)	<0.001
endocrine, nutritional, and metabolic diseases (E00–90)	3468 (14.7)	1218 (13.9)	0.08	9110 (20.5)	4509 (18.3)	<0.001
diabetes (E10–14)	2124 (9.0)	724 (8.3)	0.04	5672 (12.8)	2531 (10.3)	<0.001
diseases of the genitourinary system (N00–99)	1562 (6.6)	419 (4.8)	<0.001	5435 (12.2)	1689 (6.9)	<0.001
chronic kidney failure (N18)	576 (2.4)	91 (1.0)	<0.001	2198 (4.9)	535 (2.2)	<0.001
diseases of the digestive system (K00–93)	837 (3.5)	292 (3.3)	0.4	3543 (8.0)	1557 (6.3)	<0.001
diseases of the skin and subcutaneous tissue (L00–99)	56 (0.2)	24 (0.3)	0.6	410 (0.9)	279 (1.1)	0.01
diseases of the musculoskeletal system/connective tissue (M00–99)	662 (2.8)	676 (7.7)	<0.001	1632 (3.7)	1737 (7.1)	<0.001
diseases of the nervous system (G00–99)	330 (1.4)	331 (3.8)	<0.001	1211 (2.7)	1100 (4.5)	<0.001
lung cancer	298 (1.3)	86 (1.0)	0.04	4048 (9.1)	4533 (18.4)	<0.001
**In-hospital death**						
yes	1292 (5.5)	119 (1.4)	<0.001	4825 (10.9)	662 (2.7)	<0.001
no	22,362 (94.5)	9652 (98.6)		39,624 (89.1)	23,935 (97.3)	

**Table 3:** Characteristics of patients hospitalized with COPD by gender.

Variable	Patients Hospitalized due to COPD (n = 32,425)			Patients with COPD as a Comorbidity (n = 69,046)		
	Male n = 20,198	Female n = 12,227	*p*	Male n = 43,857	Female n = 25,189	*p*
	n (%)	n (%)		n (%)	n (%)	
**Age group**						
40–49	308 (1.5)	163 (1.3)	<0.001	692 (1.6)	321 (1.3)	<0.001
50–59	1722 (8.5)	903 (7.4)		3967 (9.0)	1876 (7.4)	
60–69	6831 (33.8)	4098 (33.5)		15,357 (35.0)	8549 (33.9)	
70–79	7085 (35.1)	4167 (34.1)		14,657 (33.4)	8636 (34.3)	
80+	4252 (21.1)	2896 (23.7)		9184 (20.9)	5807 (23.1)	
**COPD diagnosis during the admission**						
acute COPD exacerbation (J44.1)	6686 (33.1)	3946 (32.3)	0.1	6782 (15.5)	3866 (15.3)	0.7
COPD with acute lower respiratory infection (J44.0)	3577 (17.7)	2170 (17.7)	0.9	4172 (9.5)	2504 (9.9)	0.07
**Number of hospital wards**						
1	15,793 (78.2)	9231 (75.5)	<0.001	36,527 (83.3)	20,721 (82.3)	<0.001
2	4075 (20.2)	2748 (22.5)		5905 (13.5)	3509 (13.9)	
3–5	330 (1.6)	248 (2.0)		1425 (3.2)	959 (3.8)	
**First-admission hospital ward**						
internal medicine department	6471 (32.0)	4050 (33.1)	<0.001	12,844 (29.3)	7746 (30.8)	<0.001
tuberculosis and lung diseases department	2545 (12.6)	1041 (8.5)		6111 (13.9)	2736 (10.9)	
lung disease department	3334 (16.5)	1677 (13.7)		8043 (18.3)	4282 (17.0)	
emergency department	4360 (21.6)	3059 (25.0)		4830 (11.0)	2991 (11.9)	
cardiology department	73 (0.4)	72 (0.6)		2468 (5.6)	1245 (4.9)	
oncology department	10 (0.0)	11 (0.1)		1774 (4.0)	978 (3.9)	
other type of hospital ward	3405 (16.9)	2317 (18.9)		7787 (17.8)	5211 (20.7)	
**Duration of hospitalization (days)**						
0 (<24 h)	965 (4.8)	649 (5.3)	<0.001	2432 (5.3)	1333 (5.3)	<0.001
1–3	2519 (12.5)	1425 (11.7)		8473 (19.3)	4514 (17.9)	
4–7	7523 (37.2)	4241 (34.7)		14,239 (32.5)	8045 (31.9)	
8–14	5726 (28.3)	3550 (29.0)		12,060 (27.5)	7097 (28.2)	
15–21	2823 (14.0)	1880 (15.4)		3728 (8.5)	2387 (9.5)	
22–31	380 (1.9)	311 (2.5)		1588 (3.6)	970 (3.9)	
32 and more	262 (1.3)	171 (1.4)		1427 (3.3)	843 (3.3)	
**Comorbidities**						
cardiovascular diseases (I00–99)	9638 (47.7)	5820 (47.6)	0.8	29,180 (66.5)	16,759 (66.5)	0.9
hypertension (I10–15)	5238 (25.9)	3534 (28.9)	<0.001	12,015 (27.4)	8207 (32.6)	<0.001
heart failure (I50)	3705 (18.3)	2039 (16.7)	<0.001	13,875 (31.6)	7731 (30.7)	0.01
endocrine, nutritional, and metabolic diseases (E00–90)	2592 (12.8)	2094 (17.1)	<0.001	7809 (17.8)	5810 (23.1)	<0.001
diabetes (E10–14)	1687 (8.4)	1161 (9.5)	<0.001	5050 (11.5)	3153 (12.5)	<0.001
diseases of the genitourinary system (N00–99)	1500 (7.4)	481 (3.9)	<0.001	4938 (11.3)	2186 (8.7)	<0.001
chronic kidney failure (N18)	417 (2.1)	250 (2.0)	0.9	1790 (4.1)	943 (3.7)	0.03
diseases of the digestive system (K00–93)	751 (3.7)	378 (3.1)	0.003	3139 (7.2)	1961 (7.8)	0.002
diseases of the skin and subcutaneous tissue (L00–99)	43 (0.2)	37 (0.3)	0.1	446 (1.0)	243 (1.0)	0.8
diseases of the musculoskeletal system and connective tissue (M00–99)	719 (3.6)	619 (5.1)	<0.001	1702 (3.9)	1667 (6.6)	<0.001
diseases of the nervous system (G00–99)	468 (2.3)	193 (1.6)	<0.001	1532 (3.5)	779 (3.1)	0.01
lung cancer	283 (1.4)	101 (0.8)	<0.001	5659 (12.9)	2922 (11.6)	<0.001
**In-hospital death**						
yes	870 (4.3)	541 (4.4)	0.6	3474 (7.9)	2013 (8.0)	0.7
no	19,328 (95.7)	95.6 (95.6)		40,383 (92.1)	23,176 (92.0)	

**Table 4:** Factors associated with the risk of in-hospital death among patients hospitalized with COPD in Poland.

Variable	All Patients Hospitalized with COPD (n = 101,471)			Patients Hospitalized due to COPD (n = 32,425)			Patients with COPD as a Comorbidity (n = 69,046)		
	Multivariable Logistic Regression *			Multivariable Logistic Regression **			Multivariable Logistic Regression ***		
	OR	95% CI	*p*	OR	95% CI	*p*	OR	95% CI	*p*
**Age group (years)**									
40–49	1.00	Reference		1.00	Reference		1.00	Reference	
50–59	1.62	1.10–2.39	0.01	1.46	0.62–3.41	0.4	1.68	1.09–2.59	0.02
60–69	2.31	1.59–3.35	<0.001	2.26	1.01–5.10	0.04	2.37	1.56–3.59	<0.001
70–79	2.96	2.05–4.29	<0.001	2.96	1.32–6.67	0.01	3.07	2.03–4.66	<0.001
80+	4.83	3.34–7.01	<0.001	4.45	1.97–10.02	<0.001	5.19	3.42–7.88	<0.001
**Gender**									
male	0.99	0.94–1.05	0.8	0.97	0.87–1.08	0.6	1.00	0.94–1.06	0.9
female	1.00	Reference		1.00	Reference		1.00	Reference	
**Presence of cardiovascular disease (I00–99)**									
yes	1.87	1.77–1.98	<0.001	1.94	1.73–2.18	<0.001	1.65	1.54–1.77	<0.001
no	1.00	Reference		1.00	Reference		1.00	Reference	
**Presence of endocrine, nutritional ** **and metabolic disease (E00–99)**									
yes	0.80	0.75–0.85	<0.001	0.68	0.57–0.80	<0.001	0.81	0.75–0.87	<0.001
no	1.00	Reference		1.00	Reference		1.00	Reference	
**Presence of disease of the genitourinary ** **system (N00–99)**									
yes	1.55	1.44–1.67	<0.001	1.30	1.08–1.57	0.01	1.53	1.41–1.65	<0.001
no	1.00	Reference		1.00	Reference		1.00	Reference	
**Presence of disease of the digestive system ** **(K00–93)**									
yes	1.17	1.06–1.29	0.002	0.86	0.63–1.17	0.3	1.13	1.02–1.25	0.02
no	1.00	Reference		1.00	Reference		1.00	Reference	

* Multivariable logistic regression model: Cox and Snell R-Squared of 0.017 and Nagelkerke R-Squared of 0.044; ** Multivariable logistic regression model: Cox and Snell R-Squared of 0.011 and Nagelkerke R-Squared of 0.035; *** Multivariable logistic regression model: Cox and Snell R-Squared of 0.018 and Nagelkerke R-Squared of 0.043.

**Table 5:** Factors associated with the risk of prolonged hospitalization among patients hospitalized with COPD.

Variable	All Patients Hospitalized with COPD (n = 101,471)						Patients Hospitalized due to COPD (n = 32,425)					
	Hospitalization for >7 Days			Hospitalization for >10 Days			Hospitalization for >7 Days			Hospitalization for >10 Days		
	Multivariable Logistic Regression *			Multivariable Logistic Regression **			Multivariable Logistic Regression ***			Multivariable Logistic Regression ****		
	OR	95% CI	*p*	OR	95% CI	*p*	OR	95% CI	*p*	OR	95% CI	*p*
**Age group (years)**												
40–49	1.00	Reference		1.00	Reference		1.00	Reference		1.00	Reference	
50–59	1.08	0.96–1.21	0.2	1.04	0.91–1.18	0.6	1.45	1.17–1.80	<0.001	1.26	0.98–1.62	0.08
60–69	1.26	1.13–1.40	<0.001	1.21	1.07–1.37	0.003	1.99	1.63–2.44	<0.001	1.80	1.42–2.29	<0.001
70–79	1.42	1.28–1.58	<0.001	1.35	1.19–1.52	<0.001	2.19	1.79–2.69	<0.001	2.01	1.59–2.55	<0.001
80+	1.53	1.37–1.71	<0.001	1.35	1.19–1.53	<0.001	2.26	1.85–2.78	<0.001	1.88	1.48–2.40	<0.001
**Gender**												
male	1.00	Reference		1.00	Reference		1.00	Reference		1.00	Reference	
female	1.09	1.06–1.11	<0.001	1.12	1.09–1.15	<0.001	1.13	1.08–1.19	<0.001	1.20	1.14–1.26	<0.001
**Presence of at least ** **one comorbidity**												
yes	1.17	1.12–1.22	<0.001	1.10	1.05–1.15	<0.001	1.42	1.36–1.49	<0.001	1.29	1.22–1.36	<0.001
no	1.00	Reference		1.00	Reference		1.00	Reference		1.00	Reference	
**Type of admission**												
emergency	1.26	1.23–1.30	<0.001	1.00	Reference		1.00	Reference		1.00	Reference	
scheduled	1.00	Reference		1.07	1.04–1.11	<0.001	1.89	1.80–1.99	<0.001	2.90	2.75–3.06	<0.001

* Multivariable logistic regression model: Cox and Snell R-Squared of 0.008 and Nagelkerke R-Squared of 0.01; ** Multivariable logistic regression model: Cox and Snell R-Squared of 0.002 and Nagelkerke R-Squared of 0.003; *** Multivariable logistic regression model: Cox and Snell R-Squared of 0.029 and Nagelkerke R-Squared of 0.038; **** Multivariable logistic regression model: Cox and Snell R-Squared of 0.051 and Nagelkerke R-Squared of 0.074.

## Data Availability

Data are available from the corresponding author on reasonable request.
